# GTn Repeat Microsatellite Instability in Uterine Fibroids

**DOI:** 10.3389/fgene.2019.00810

**Published:** 2019-09-20

**Authors:** Bineta Kénémé, Mbacké Sembène

**Affiliations:** ^1^GenGesPop, Cheikh Anta Diop University, Animal Biology, Dakar-Fann, Senegal; ^2^Biopass, IRD, Dakar-Bel Air, Senegal

**Keywords:** uterine fibroid, COL1A2 polymorphism, risk factors, Senegal, microsatellite genetic marker

## Abstract

**Background:** *Type I collagen* is a triple helix structure with two α1 and one α2 chains. Coordinated biosynthesis of α1 and α2 subunits is very important for tissue morphogenesis, growth, and repair. In contrast, abnormal deposition in response to proinflammatory cytokines is associated with organ dysfunction. In humans, *COL1A2* contains two microsatellite loci: one located at the 5’-flanking region is composed of poly CA and poly CG; the other located in the 1st intron is constituted of poly GT. Expression of *COL1A2* has been noted in gastric cancer and was positively correlated with degree of invasion and metastases. But no genetic study taking into account polymorphism of *COL1A2* in uterine fibroids has been undertaken.

**Methods:** In this study, repeated dinucleotide **GT** **_n_** of intron 1 ***COL1A2*** was highlighted in 55 patients with uterine fibroids (UF). Clinical and pathological data were obtained from patient’s records, and other parameters were recorded. Mutation Surveyor version 5.0.1, DnaSP version 5.10, MEGA version 7.0.26, and Arlequin version 3.5.1.3 were used to determine genetics parameters. To estimate genetic variation according to epidemiological parameters, index of genetic differentiation (Fst) and genetic structure (AMOVA) were determined with Arlequin version.

**Results:** Based on reference microsatellite pattern **(GT)** **_14_** **CT(GT)** **_3_** **CT(GT)** **_3_**, 15 haplotypes were found. Among the 15 haplotypes, 12 have mutation at position **2284C > G** and 7 at position **2292C > G**. Insertions of repeated dinucleotide GT_n_ were found on three haplotypes against eight haplotypes in which they are deletions. Intron 1 of *COL1A2* gene exhibits high genetic diversity in uterine fibroids with 35.34% polymorphic sites, 95.74% of which were parsimoniously variable and an average number of nucleotide difference of 10.442, which reflects an important genetic variability. According to epidemiological parameters, our results showed, for the first time, a genetic structuring of uterine fibroids according to ethnicity, marital status, use of contraception, diet, and physical activity, beyond confirming the involvement dinucleotide length polymorphism GT_n_ in occurrence of uterine fibroids in Senegalese women.

**Conclusion:** Results obtained open up avenues for understanding the mechanisms involved in the racial variation in the prevalence of uterine fibroids as well as the predisposing factors.

## Introduction

Uterine fibroids (UF), more commonly known as myomas or uterine leiomyomas, are the most common benign tumors of female reproductive organs. They are associated with significant morbidity and therefore constitute a real public health problem. UF, which are highly variable within uterus, develop at the expense of smooth muscle and are often separated from the myometrium by a pseudocapsule associated with connective tissue condensation (Audebert, 1990). Heterogeneity of UF localization and their progression in the same patient illustrate the complex biological mechanism involved in their development. Clinically, UF are firm, stiff nodular tumours, a fact confirmed by biomechanical studies. Proteins of extracellular matrix, especially interstitial collagens, are responsible for this property of “firmness” and mechanical strength of tissue. Indeed, UF have an accumulation of altered collagens and different amounts of glycosaminoglycans and a proliferation of cells, which is by definition a fibrosis. A complete understanding of the role of extracellular matrix proteins, in particular collagen, and their effect on the growth and development of UF becomes an important issue for elucidating molecular mechanisms involved in their etiology.

Located on chromosome 7, *COL1A2* is an essential component of matrix tissue. It is predominantly produced by mesenchymal cells such as fibroblasts, osteoblasts, and smooth muscle cells. Transcription of *COL1A2* is under control of a regulatory complex that includes several DNA elements and several trans-activating factors. During the last two decades, *type I alpha chain collagen 2* (*COL1A2*) has been considered as an informative model for studying principles that govern the control of extracellular matrix transcription for normal and fibrotic tissues (Kirkland, 2009; Krasny et al., 2010; Trojonowska, 2002; Yasul et al., 2004). In humans, *COL1A2* contains two microsatellite loci: one located at the 5’-flanking region of the gene is composed of poly CA and poly CG; the other located in the 1st intron is constituted of poly GT. In a study led by Akai et al. (1999), it has been shown that complete transcription of *COL1A2* gene is regulated by these repeated dinucleotides. Analysis of polymorphism in these two regions indicates that these two sequences show a variation in their repetition number, suggesting that these dinucleotides constitute microsatellites. Lei et al. (2005) hypothesized that GT_n_ polymorphism triggers transcription of the gene, and variation in the number of repetitions can partly be responsible for the difference in transcriptional activity. In this study, we evaluate instability of repeated dinucleotide **GT** **_n_** in Senegalese patients with UF.

## Materials and Methods

### Clinical Sampling

Tumor tissue samples were collected from 55 patients with UF (from Military Hospital of Ouakam and General Hospital of Grand Yoff). Clinical and pathological data were recorded including age, ethnicity, age at menarche, marital status, number of pregnancies, number of childbirth, hormonal contraception, diet, and physical activity (**Table 1**). None of the patients surveyed claimed to consuming alcohol and using tobacco, which is why these factors are not included in this study.

**Table 1:** Clinical and pathological characteristics of 55 cases analyzed.

Epidemiological factors	Number of patients (%)
**Age (n = 36)**	
≤35	11 (30.55%)
]35–45]	18 (50%)
> 45	7 (19.45%)
**Ethnicity (n = 39)**	
Wolof	13 (33.33%)
Sérère	4 (10.26%)
Lébou	7 (17.95%)
Bambara	3 (7.69%)
Diola	5 (12.82%)
Alpulaar	7 (17.95%)
**Marital status (n = 31)**	
Single	8 (25.80%)
Married	20 (64.52%)
Divorced	3 (9.68%)
**Age at menarche (n = 18)**	
≤12	1 (5.56%)
]12–15]	13 (72.22%)
> 15	4 (22.22%)
**Number of pregnancies (n = 31)**	
0	20 (64.51%)
I	4 (12.91%)
II	4 (12.91%)
III	1 (3.22%)
> III	2 (6.45%)
**Number of childbirth (n = 33)**	
0	23 (69.70%)
I	7 (21.21%)
II	1 (3.03%)
III	2 (6.06%)
> III	0 (0%)
**Hormonal contraception (n = 23)**	
**Yes**	2 (8.69%)
**No**	21 (91.31)
**Diet (n = 23)**	
Meat preference	7 (30.43%)
Vegetarian preference	6 (26.09%)
No preference	10 (43.48%)
**Physical activity (n = 23)**	
Yes	5 (21.74%)
No	18 (78.26%)

### DNA Extraction, Amplification, and Sequencing of Intron 1 *COL1A2* Gene

Total DNA of each sample was extracted using Qiagen protocol (Qiagen Dneasy Tissue kit). After extraction, repeated dinucleotide **GT** **_n_** were amplified using forward 5’-TGTCTACCACTGCATAATTTC-3 and reverse 5’-AATATGAACTCGGTAATGTGA-3’ primers (Lei et al., 2005). The 35 cycle PCR for *COL1A2* intron 1 amplification was carried out using 4 μl of human genomic DNA in a 50 μl reaction mixture, which contained 0.1 μl of Taq DNA polymerase, 2.5 μl of forward and reverse primers, 1 μl of magnesium chloride, 2 μl of mix dNTPs, and 5 μl of 10X ammonium sulfate buffer. Thermal cycle conditions for amplification PCR consisted of 1st step-3 min cycle of initial denaturation at a temperature of 94°C, followed by 2nd step consisting of 35 cycles each of 45 s of denaturation at 94°C, annealing at 60°C/1min and primer extension at 72°C/1 min, and 3rd step: final extension or polymerization at 72°C for 10 min. After PCR reaction, all products were electrophoresed on 1.5% agarose gel, followed by its analysis in an UVitec Gel Documentation system for imaging the gel and to determine the amplicon lengths. Sequencing reactions were performed in a thermal cycler MJ Research PTC-225 Peltier type with ABI PRISM BigDye TM Terminator Cycle kit. Each sample was sequenced using forward primer. Fluorescent fragments were purified with the BigDye Xterminator purification protocol. The samples were suspended in distilled water and subjected to electrophoresis in 3730xl ABI sequencer (Applied Biosystems).

### Molecular Analysis

To determine length polymorphism of dinucleotide **GT** **_n_** of intron 1 *COL1A2* gene, the raw sequencing data were submitted to Mutation Surveyor software version 5.0.1 (www.softgenetics.com). This program can directly compare chromatograms with genomic DNA of reference sequence of *COL1A2* (NT_007933_94023373). Alignment of the sequences was carried out using BioEdit software version 8.0.5 and ClustalW algorithm (Thompson et al., 1994). Sequences obtained (Hall, 1999) were thoroughly checked, cleaned, and aligned to identify homologies among sites, and also to perform other phylogenetic analysis including the determination of variability index and genetic diversity as well as the parameters of genetic differentiation. Genetic variability parameters (number of polymorphic sites, total number of haplotype, average number of nucleotide difference K) were obtained through DnaSP 5.10 software (Librado and Rozas, 2009) and MEGA 7.0.26 (Kumar et al., 2016). To estimate genetic variation according to epidemiological parameters, the factor of genetic differentiation (Fst) and the analysis of molecular variance (AMOVA) were determined with Arlequin software version 3.5.1.3 (Excoffier and Lischer, 2010). Values of P less than 0.05 are considered significant at a 5% confidence interval.

## Results

### Mutations Status of Microsatellite GT_n_

**Figure 1:**
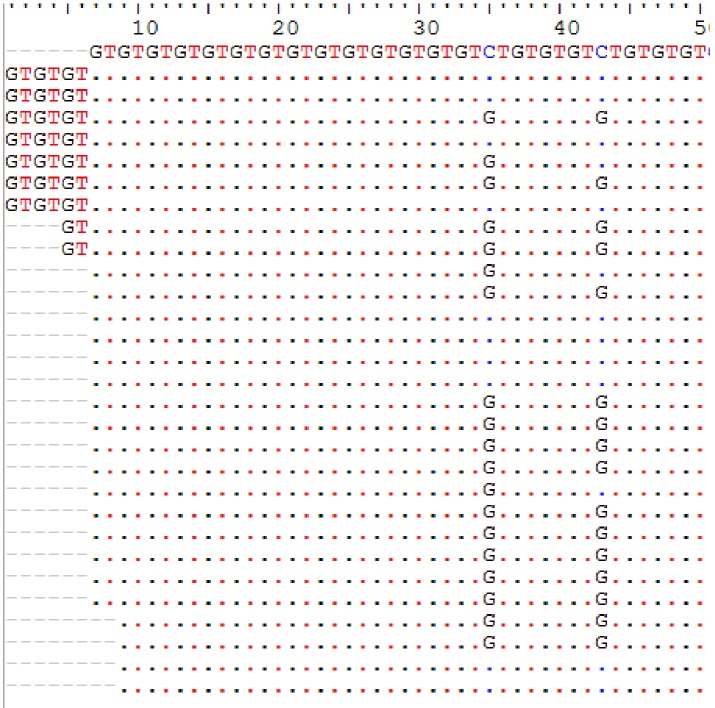
Microsatellite GT_n_ length and pattern polymorphism in uterine fibroids.

*COL1A2* was sequenced in 55 tumour tissues. Of these sequences, five were removed from the genetic analysis because of a strong polymorphism. Based on the microsatellite reference pattern in the form (GT)_14_CT(GT)_3_CT(GT)_3_, 15 haplotypes were found in 50 Senegalese women with UF (**Table 2**). These haplotypes indicate a variation in GT repetition number ranging from 13 to 25 (**Figure 1**).

**Table 2:** Length and pattern polymorphism of repeated dinucleotide GT_n_ of intron 1 *COL1A2* gene in uterine fibroids.

Haplotype	Number (%)	Microsatellite pattern	Variants
**H1**	5 (10%)	(GT) _14_CT(GT)_3_CT(GT) _3_	Wide type
**H2**	4 (8%)	(GT)_17_CT(GT)_3_CT(GT)_3_	2276_2281_InsGTGTGT
**H3**	2 (4%)	GT_25_	2276_2281_InsGTGTGT; 2284C > G; 2292C > G
**H4**	1 (2%)	(GT)_21_CT(GT)_3_	2276_2281_InsGTGTGT; 2284C > G
**H5**	2 (4%)	GT_23_	2281-2282_InsGT; 2284C > G; 2292C > G
**H6**	2 (4%)	(GT)_18_CT(GT)_3_	2284C > G
**H7**	10 (20%)	GT_22_	2284C > G; 2292C > G
**H8**	6 (12%)	(GT_21_)	2282-2283_DelGT; 2284C > G; 2292C > G
**H9**	2 (4%)	(GT)_13_CT(GT)_3_CT(GT)_3_	2282-2283_DelGT
**H10**	1 (2%)	(GT)_17_CT(GT)_3_	2282-2283_DelGT; 2284C > G
**H11**	7 (14%)	(GT)_16_CT(GT)_3_	2280-2283_DelGTGT; 2284C > G
**H12**	4 (8%)	GT_20_	2280-2283_DelGTGT; 2284C > G; 2292C > G
**H13**	1 (2%)	GT_19_	2276-2281_DelGTGTGT; 2284C > G; 2292C > G
**H14**	2 (4%)	(GT)_15_CT(GT)_3_	2276-2281_DelGTGTGT; 2284C > G
**H15**	1 (2%)	GT_18_	2274-2281_DelGTGTGTGT; 2284C > G; 2292C > G

Ins, insertion; Del, deletion.

**Figure 2:**
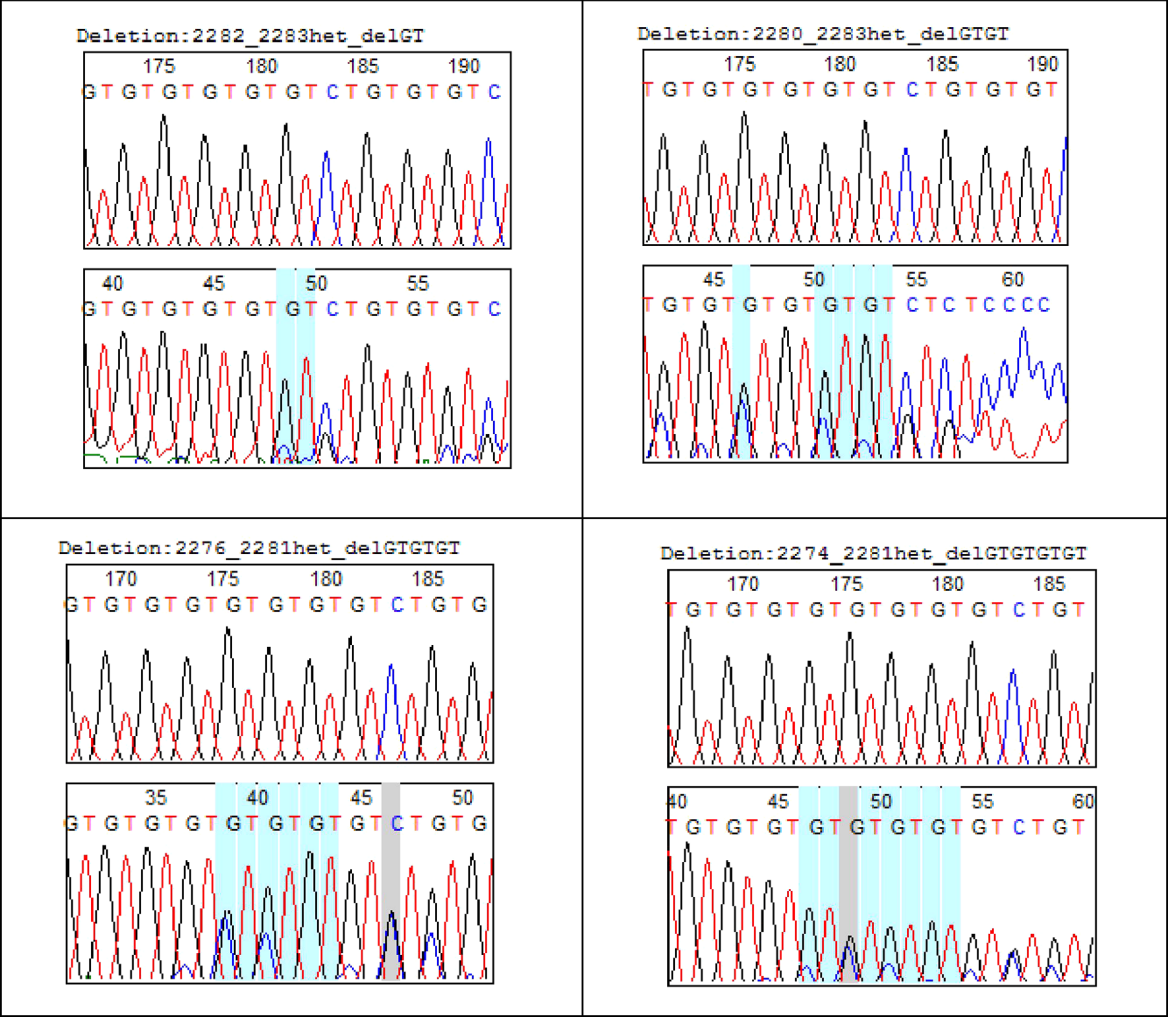
GT_n_ *COLLA2* deletion in uterine fibroids.

Of the 15 haplotypes, 12 have mutation at position **2284C > G** (first site that interrupts GT dinucleotide repeat) and 7 have mutation at **2292C > G** (2nd site that interrupts GT dinucleotide repeat). **Insertions** of repeated dinucleotide GT_n_ were found on three haplotypes (microsatellite elongation) compared to eight haplotypes in which they were **deletions** (microsatellite shortening). Haplotype 7 representing 20% of the haplotypes was characterized by the presence of two types of transversions **2284C > G** and **2292C > G**. Haplotypes 11 (14%) and 8 (12%) were respectively characterized by deletions at position **2280-2283_DelGTGT** and **2282-2283_DelGT** (**Table 2**). Some microsatellite length polymorphisms of **GT** **_n_** *COL1A2* were summarized in **Figure 2**.

### *COL1A2* Intron 1 Polymorphisms

Intron 1 of *COL1A2* gene exhibits high genetic diversity in UF with 35.34% polymorphic sites, 95.74% of which were parsimoniously variable. Average number of nucleotide differences was 10.442 (**Table 3**). The high haplotypic diversity (Hd = 0.9984) and the low nucleotide diversity (Pi = 0.0877) showed a rapid evolution of microsatellite polymorphism in UF in Senegalese women.

**Table 3:** Index of variability and genetic diversity of intron 1 *COL1A2* in fibroid cases.

Variability index			
Parameters		Number	**Percentage**
Number of sequences		50	
Number of sites		133	
Monomorphic sites		86	64.66%
Polymorphic sites		47	35.34%
Singleton variable sites		2	4.26%
Parsimony informative sites		45	95.74%
Average number of nucleotide differences (k)		10.442	
**Genetic diversity index**			
**Pi ± variance**	0.0877 ± 0.00002		
**Hd ± variance**	0.9984 ± 0.00003		

Hd, haplotypic diversity; Pi, nucleotide diversity.

### Microsatellite GT_n_ Instability and Genetic Differentiation

Depending on epidemiological parameters studied, only repeated dinucleotide is taken into account in analysis. This allows us to highlight the role of the length polymorphism of intron 1 *COL1A2*. Results obtained show a variable expressivity of dinucleotide GT_n_ in Senegalese women with UF (**Table 4**). For age parameter, tumoral tissues were genetically more different in women under 35 (Fst = 0.04237) and those over 45 (Fst = 0.06574) compared to women aged 35–45 years (Fst = 0.03152). This differentiation is more significant between the two extremes (under 35 and over 45). This heterogeneity of repeated dinucleotide GT_n_ polymorphism is more noticeable among women of Bambara, Sérère, Lébou and Alpulaar ethnic groups, UF being genetically homogeneous in Wolof and Diola women. Strong genetic differentiation was noted between Wolof and Alpulaar.

**Table 4:** Degree of genetic differentiation of repeated dinucleotide GT_n_ of *COL1A2* gene in relation to the epidemiological parameters studied.

Epidemiological parameters	Genetic differentiation (Fst)		
Groups Sub-groups	Within sub-groups	Between sub-groups	
Age	**Fst**	**Between sub-groups**	**Fst (P-value)**
≤35	0.04237	≤35 &]35–45]	0.01274 (0.32422)
]35–45]	0.03152	≤35 & >45	0.20818 (0.07812)
> 45	0.06574	]35–45] & >45	−0.02089 (0.46582)
**Ethnicity**			
Wolof	0.00180	Wolof & Sérère	0.15238 (0.17773)
Sérère	0.20123	Wolof & Lébou	0.06714 (0.29785)
Lébou	0.14805	Wolof & Bambara	−0.03106 (0.99902)
Bambara	0.30276	Wolof & Diola	0.10515 (0.19531)
Diola	−0.00183	Wolof & Alpulaar	0.18605 (0.07324)
Alpulaar	0.11805	Sérère & Lébou	−0.16129 (0.76562)
		Sérère et Bambara	0.06667 (0.99902)
		Sérère & Diola	0.15152 (0.15918)
		Sérère & Alpulaar	0.12513 (0.12891)
		Lébou & Bambara	−0.33333 (0.99902)
		Lébou & Diola	−0.09091 (0.60938)
		Lébou & Alpulaar	−0.08691 (0.72852)
		Bambara & Diola	−0.40000 (0.99902)
		Bambara & Alpulaar	−0.52941 (0.99902)
		Diola & Alpulaar	0.13754 (0.11523)
**Marital status**			
Single	0.08964	Single & Married	0.03216 (0.24805)
Married	0.10771	Single & Divorcée	0.39683 (0.99902)
Divorced	0.23556	Married & Divorced	0.38444 (0.99902)
**Age at menarche**			
≤12	0.20408	≤12 &]12 – 15]	−0.45799 (0.91992)
]12 – 15]	−0.14031	≤12 & >15	0.00000 (0.39453)
>15	−0.08291	] 12 – 15] & >15	−0.01850 (0.48242)
**Number of pregnancies**			
0	0.00988	0 & I	−0.06950 (0.70508)
I	0.02840	0 & II	0.00284 (0.38184)
II	−0.11721	0 & III	−0.01720 (0.49707)
III	0.34321	0 & > III	0.42177 (0.99902)
> III	0.34321	I & II	−0.35429 (0.80273)
		I & III	−0.10092 (0.70703)
		I & > III	−0.17647 (0.99902)
		II & III	0.13333 (0.31641)
		II & > III	−1.00000 (0.99902)
		III & > III	1.00000 (0.99902)
**Number of childbirth**			
0	−0.06871	0 & I	0.07169 (0.18652)
I	−0.13999	0 & II	−0.41520 (0.99902)
II	0.26463	0 & III	−0.41520 (0.99902)
III	0.26463	I & II	−0.16049 (0.99902)
		I & III	−0.16049 (0.99902)
		II & III	0.00000 (0.99902)
**Hormonal contraception**			
Yes	0,17376	Yes & No	0.24883 (0.07129)
No	−0,07280		
**Diet**			
1 Meat preference	0.01042	1 & 2	0.17374 (0.07520)
2 Vegetarian preference	−0.01543	1 & 3	−0.11892 (0.92285)
3 No preference	0.01406	2 & 3	0.20431 (0.04785)
**Physical activity**			
Yes	0.14435	Yes & No	0.15107 (0.06445)
No	0.07661		

According to marital status, UF seem to have the same genetic characteristics in single women (Fst = 0.08964), unlike married women (Fst = 0.10771) and divorced women (Fst = 0.23556), where there is a strong genetic differentiation within each group. However, no statistically significant differentiation is noted between these groups.

For the age at menarche variable, only one woman who reported having menarche before the age of 12 had a different haplotype than the remaining 17 women on which age of menarche data was available. Further investigation with a larger patient cohort is required to determine the significance of this observation. As for the number of pregnancies, no statistically significant differentiation is noted between the sub-groups, but nevertheless, we notice a strong genetic differentiation in three women with three and more than three pregnancies. It is the same for the number of childbirth. Compared to hormonal contraception on the one hand and physical activity on the other hand, we noted an important genetic differentiation between sub-groups.

Polymorphism of repeated dinucleotide GT_n_ was genetically different within women who have a meat preference and those who have no food preference. Women who are preferably vegetarians were genetically homogeneous (**Table 4**).

### Microsatellite GT_n_ Instability and Molecular Variance Analysis

The Fst values are further explained by molecular variance analysis (**Table 5**). Repeated dinucleotide GT_n_ analysis showed that UF are genetically structured according to ethnicity (p = 0.03421*), marital status (p = 0.00782**), hormonal contraception (p = 0.00098***), dietary preference (p = 0.04301*), and physical activity (p = 0.00684***). In other words, molecular mechanisms of *COL1A2* involved in etiology of UF in Senegalese women are modulated by risk factors such as ethnicity, marital status, hormonal contraception, diet, and physical activity. Indeed, the polymorphism of the repeated dinucleotide GT_n_ of intron 1 *COL1A2* in UF is explained to:

- 8.83% by differentiation between women of different ethnic groups;
- 10.57% by a differentiation between women according to their marital status;
- 24.88% by differentiation according to whether or not use of hormonal contraception;
- 7.94% by dietary preference; and
- 15.10% by a differentiation according to physical activity.

**Table 5:** Genetic structuring of GT_n_ *COL1A2* according to epidemiological parameters.

Epidemiological parameters	Source of variation	Percentage of variation	Fst (P-value)
Age	Within sub-groups	96.03862	0.03961 (0.06843)
	Between sub-groups	3.96138	
Ethnicity	Within sub-groups	91.16560	0.08834 (0.03421)
	Between sub-groups	8.83440	
Marital status	Within sub-groups	89.42471	0.10575 (0.00782)
	Between sub-groups	10.57529	
Age at menarche	Within sub-groups	108.72040	−0.08720 (0.88368)
	Between sub-groups	−8.72040	
Number of pregnancies	Within sub-groups	95.80665	0.04193 (0.21994)
	Between sub-groups	4.19335	
Number of childbirth	Within sub-groups	105.40304	−0.05403 (0.79570)
	Between sub-groups	−5.40304	
Hormonal contraception	Within sub-groups	75.11658	0.24883 (0.00098)
	Between sub-groups	24.88342	
Diet	Within sub-groups	92.05111	0.07949 (0.04301)
	Between sub-groups	7.94889	
Physical activity	Within sub-groups	84.89311	0.15107 (0.00684)
	Between sub-groups	15.10689	

Since there is no multivariate analysis and the sample sizes are small for some of these variables, more research is needed to highlight these results.

## Discussion

### *COL1A2* Polymorphisms in Uterine Fibroids

Located on chromosome 7, *COL1A2* is an essential component of the tissue matrix. It is predominantly produced by mesenchymal cells such as fibroblasts, osteoblasts, and smooth muscle cells (Rossert et al., 2000). Transcription of *COL1A2* is under control of a regulatory complex that includes several DNA elements and several trans-activating factors. In humans, *COL1A2* contains two microsatellite loci: one located at the 5’-flanking region of the gene is composed of poly CA and poly CG; the other located in the 1st intron is constituted of poly GT. In this study, microsatellite polymorphism GT_n_ of intron 1 *COL1A2* was highlighted in cases of UF in Senegalese women. Based on microsatellite reference pattern that is (GT)_14_(CT)(GT)_3_(CT)(GT)_3_, 15 haplotypes were found. These haplotypes indicate a variation in number of GT repeats ranging from 13 to 25. Of the 15 haplotypes, 12 have the 2284C > G mutation and 7 have the 2292C > G mutation. Insertions of the dinucleotide GT_n_ were found on three haplotypes (microsatellite elongation) compared to eight haplotypes in which they were deletions (microsatellite shortening). This suggests an altered mechanism of the role of *COL1A2* gene in UF. Indeed, in a study led by Lei et al. (2005), it has been hypothesized that intron 1 GT_n_ polymorphism triggers transcription of gene and variation in number of repeats may be partly responsible for the difference in transcriptional activity. In addition, about 200 different chromosomal abnormalities have been described in UF including long-arm translocations of chromosome 7 occurring in about 17% of karyotypically abnormal UF (Sandberg, 2005). In contrast to normal tissues where collagen is organized into long, thin, wavy fibrils parallel to the epithelial boundary, collagen fibrils in the tumor stroma are thicker and shorter (Cho et al., 2015). In epithelial ovarian cancer, collagenous pathways perpendicular to the epithelial boundary have been observed (Adur et al., 2014).

Intron 1 of *COL1A2* gene exhibits high genetic diversity in UF with 35.34% polymorphic sites, 95.74% of which are parsimoniously variable and an average number of nucleotide differences of 10.442, which reflects an important genetic variability. This could be explained by the fact that compared to the myometrium, in UF, not only expression of collagen genes increases (Stewart et al., 1994), but also amount of mature reticulated collagen protein is increased and the more important is modified (Leppert et al., 2004). UF are firm, stiff nodular tumors, a fact understood by all clinicians and confirmed by biomechanical studies (Rogers et al., 2008; Jayes et al., 2013). Extracellular matrix (ECM) proteins, including interstitial collagens, are responsible for this property of “firmness” and mechanical strength of tissues. ECM is a structure that has a supporting role, but on the other hand, it provides signals to cells that determines their behavior. The role of ECM and mechanotransduction as an important signaling factor in human uterus is just beginning to be appreciated. ECM is not just substance surrounding cells, but rigidity compresses cells or stretches them into signals converted into chemical changes, depending on amount of collagen, crosslinking and hydration, as well as other components of ECM. Since connective tissue integrity, architecture, and function result from specific interactions between collagen and other components of ECM, the presence of abnormal collagen chains may have a strong influence on metabolism of non-collagenic components (Tenni et al., 1988). According to study by Hauptman et al. (2018) in colorectal cancers, the results showed that 9 of 16 genes that show differential expression in carcinomas compared to adenomas are components of ECM. Among these components, two collagen type I proteins (COL1A1, COL1A2) are significantly over-regulated in cancerous tissues compared to normal tissues. Studies on cell lines suggest that type I collagen adhesion promotes intracellular signaling pathways.

### Microsatellite GT_n_ Instability in Uterine Fibroids: Correlation With Epidemiological Parameters

#### Ethnicity

In addition to great variability, repeated dinucleotide GT_n_ of intron 1 *COL1A2* exhibits heterogeneity given to clinico-pathological parameters in women with UF. Heterogeneity of predisposing factors involved in UF illustrates the complex biological mechanism involved in their development. This suggests the involvement of several molecular mechanisms in occurrence of UF. Prospective studies with larger number of samples would strengthen the correlation observed in the current study. Epidemiological data have mentioned racial disparity in occurrence of UF. Ethnicity has a major influence on development and clinical severity of UF. African-American women develop UF at higher frequency and with more severe symptoms. Hispanic women have an intermediate disease profile, and Caucasian women are the least severely affected ethnic group (Velebil et al., 1995; Baird et al., 2003; Wise et al., 2012). It appears that increased incidence and severity of disease in African-American women may be due to a combination of specific genetic and environmental factors that are not independent risk factors for the disease (Commandeur et al., 2015). In this study, we took ethnicity into account, although these women are all black. UF are genetically heterogeneous in Bambara, Sérère, Lébou, and Alpulaar and more homogeneous in Diola and Wolof. In a study conducted by Thiaw (2018) on ethnic diversity of Senegalese population (unpublished data), analysis of GT_n_ pattern polymorphism shows a genetic differentiation between Diola and Wolof compared to other ethnic groups. This differentiation may explain in part 8.83% of genetic structure of *COL1A2* observed in UF by ethnicity.

#### Marital Status

Genetic differentiation observed (10.57%) is also explained by the differentiation between women according to their marital status; greater differentiation is observed among married and divorced women compared to single. This could be explained by the difference in hormonal status in these women. Studies of Barrett et al. (2015) on ovarian steroid status in marital status showed that estradiol was higher among married women than among unmarried women (β = 0.19, 95% CI: 0.02–0.36) as well as progesterone (β = 0.19, 95% CI: 0.01–0.39). In addition, many clinical observations indicate that the development of UF is related to hormonal status (Ross et al., 1986). For example, UF do not occur in prepubertal women and are rarely seen in adolescent girls (Fields and Neinstein, 1996).

#### Hormonal Contraception

Our results also indicated that 24.88% of differentiation observed in cases of UF is explained by a differentiation following hormonal contraception use. Relationship between oral contraceptives and UF has been largely elucidated. But epidemiological data between contraceptive use and UF seems controversial. Published studies show a reduction or absence of risk between oral contraceptives use combined with appearance of UF (Berisavac et al., 2009). One study has shown that oral contraception may play a role in development of UF. Others have found no association between occurrence of UF and use of contraception (Parazzini et al., 1992).

#### Diet

In relation to diet, a positive correlation was noted between dietary preference and genetic expression of *COL1A2* in UF (7.94% of genetic differentiation). Genetic differentiation is more observed in patients with meat preference. Recently, Wise and Laughlin-Tommaso (2016) published results on relationship between dietary fat intake and UF risk in African-American women, confirming an increased risk associated with consumption of omega-3 fatty acids long chain. They validated hypothesis that a diet rich in fruits and vegetables reduced risk. According to studies of Chiaffarino (1999), women with UF consume beef, other red meats, and ham more frequently and have less frequent consumption of green vegetables, fruits, and fish. Multivariate rib ratios were 1.7 for beef and other red meats, 1.3 for ham, and 0.8 for fruit consumption. Limitation of this current diet study is the lack of data on total energy intake because information was collected only on the frequency of vegetable consumption compared to red meat and in interviews with patients. Further research would be interesting to evaluate the effect of fat intake on uterine fibroids biology.

#### Physical Activity

There have been few studies on effect of physical activity on risk of developing UF. Nevertheless, our results showed a genetic structuring of UF according to practice or not of sport (15.10% of genetic differentiation). Since this is a modifiable factor, more research is needed to evaluate effects of physical activity on UF biology.

## Conclusion

Results obtained show, for the first time, a genetic structuring of UF according to ethnicity, marital status, use of contraception, diet, and physical activity, beyond confirming the involvement of *COL1A2* gene, in particular dinucleotide length polymorphism GT_n_ in occurrence of UF in Senegalese women. In addition to this, results obtained open up avenues for understanding the mechanisms involved in racial variation in the prevalence of UF as well as the predisposing factors. Given the admitted results, it is clear that more research is needed to determine risk factors associated with appearance and growth of UF, as they cause significant morbidity and affect quality of life. A clear overview of the epidemiology of UF has not yet been realized and future research on modifiable risk factors such as vegetarian diet, contraception, physical activity, among others could inform the prevention of myomas and provide new non-surgical approaches to treatment.

## Ethics Statement

This study was carried out in accordance with the recommendations of World Medical Association’s Declaration of Helsinki. The protocol was approved by the Institutional Ethics Committee on Human Research of Cheikh Anta Diop University (Reference: Protocol 0267/2017/CER/UCAD). All subjects gave written informed consent according to a standardized form.

## Author Contributions

BK performed molecular analysis, organized the database, performed data analysis, and wrote the first draft of the manuscript. MS contributed to conception and design of the study, revised the manuscript, and read and approved the submitted version.

## Conflict of Interest Statement

The authors declare that the research was conducted in the absence of any commercial or financial relationships that could be construed as a potential conflict of interest.
